# Cross‐Cultural Adaptation and Psychometric Evaluation of the DoFEL‐R for Early Detection of Alzheimer's Related Functional Decline in Croatia

**DOI:** 10.1002/hsr2.71442

**Published:** 2026-01-03

**Authors:** Freddie O'Donald, Mario A Parra, Clara Calia

**Affiliations:** ^1^ School of Health in Social Science University of Edinburgh Edinburgh UK; ^2^ Department of Psychology NHS Tayside Dundee UK; ^3^ School of Psychological Sciences and Health University of Strathclyde Glasgow UK

**Keywords:** Alzheimer's disease, cross‐cultural adaptation, early detection, functional assessment, memory binding, psychometric validation

## Abstract

**Background and Aims:**

Early‐stage Alzheimer's disease (AD) is often associated with subtle functional changes that may go undetected by conventional assessment tools. The Details of Functions of Everyday Life – Revised (DoFEL‐R) was developed to address this gap by embedding updated theoretical understandings from memory binding into the assessment of everyday tasks. This study aimed to translate, cross‐culturally adapt, and conduct a preliminary validation of the DoFEL‐R for use with Croatian‐speaking older adults.

**Methods:**

Following the guidelines of the International Test Commission (ITC), the adaptation process included forward and back translation, a Delphi expert panel, cognitive interviews, and pilot testing. A total of 263 community‐dwelling older adults completed the Croatian DoFEL‐R, with fifty‐six participating in a 2‐week follow‐up to assess test‐retest reliability.

**Results:**

Exploratory factor analysis supported a two‐factor structure aligned with relational and conjunctive binding, consistent with the original theoretical framework. The scale showed strong internal consistency (Cronbach's *α* = 0.89) and excellent test‐retest reliability (ICC = 0.91). Cognitive interviews indicated good face validity and cultural appropriateness of the adapted items.

**Conclusion:**

These findings support the Croatian DoFEL‐R as a valid and reliable tool for identifying early functional decline indicative of AD‐related dementia in older adults. Its use may enhance early detection of preclinical AD and inform clinical assessment practices in Croatia and similar cultural contexts. However, the sample's relatively high educational and digital literacy levels may limit generalisability, and further research is needed to confirm applicability across more diverse populations.

## 1 Introduction

Early‐stage Alzheimer's disease (AD) is associated with subtle cognitive and functional impairments that often go undetected by conventional assessment tools [1]. While mild cognitive impairment (MCI) is often considered a prodromal phase of AD, functional difficulties may already be present at this stage and offer diagnostic value [2]. However, available tools frequently lack sensitivity to these early changes and are rarely grounded in cognitive theory, such as memory binding, which is increasingly recognised as an early marker of AD‐related pathology [3]. The Details of Function of Everyday Life – Revised (DoFEL‐R) scale was developed to address these limitations by embedding principles of relational and conjunctive memory binding into a functional assessment framework [4].

This study presents the translation, cross‐cultural adaptation, and preliminary psychometric validation of the DoFEL‐R for use with Croatian‐speaking older adults. Guided by the [5], the adaptation process incorporated a Delphi review, pilot testing, and cognitive interviews. This study aims to assess the face validity, factor structure, internal consistency, and test‐retest reliability of the Croatian DoFEL‐R to establish its suitability as a culturally relevant tool for identifying early functional decline potentially linked to preclinical AD stages.

## 2 Methods

This study received ethical approval from the University of Edinburgh, School of Health in Social Science Research Ethics Committee. All participants provided informed consent in accordance with the Declaration of Helsinki.

### 2.1 Translation and Cross‐Cultural Adaptation

The Details of Functions of Everyday Life – Revised (DoFEL‐R) questionnaire was translated and adapted from UK English into Croatian following the International Test Commission (ITC) guidelines for test adaptation (International Test Commission, 2017). The process included forward and back translation, expert review using a Delphi method [6], pilot testing, and psychometric evaluation.

#### 2.1.1 Translation Procedure

Two independent forward translations into Croatian were completed by bilingual native Croatian speakers with high English fluency and familiarity with healthcare and psychological terminology. The lead researcher reconciled the translations into a single version. Then, two independent back‐translations into English were conducted by native English speakers fluent in Croatian who were blind to the original questionnaire. Discrepancies were discussed and resolved to ensure semantic and conceptual equivalence with the source version.

#### 2.1.2 Expert Panel Review (Delphi Method)

Through purposive sampling, an expert panel of six participants (a neuropsychologist, two geriatricians, two older adults with an interest in psychology and dementia care, and a dementia nurse specialist) was formed. Based on Teig et al. [6], a two‐step Delphi process was conducted using Qualtrics to administer surveys and collate responses.

Panel members independently evaluated the translated items for clarity, cultural relevance, and conceptual equivalence. Responses were anonymised across rounds to reduce bias and to ensure equal input. Additionally, several cross‐cultural adaptations were made following panel consensus, including:

- Reframing certain items to reflect the use of digital payment methods and messaging apps (e.g., WhatsApp, Viber) in daily functioning.
- Incorporating public transport use alongside driving.
- Accounting for traditional cooking practices in Croatia.
- Adjusting items to reflect family‐centred social structures and the use of herbal remedies alongside conventional medications.

#### 2.1.3 Pilot Testing and Cognitive Interviews

To assess face validity, the intermediate Croatian version of the DoFEL‐R was piloted with a convenience sample of four older adults (67–84 years old; 50% female, with a mean education of 15.7 years). Participants completed the questionnaire and participated in cognitive interviews to identify unclear or culturally incongruent items [7]. Based on feedback, minor wording clarifications were made. No significant issues were identified.

### 2.2 Psychometric Evaluation

The psychometric evaluation aimed to assess the factor structure, internal consistency, and test‐retest reliability of the Croatian version of the DoFEL‐R in a sample of community‐dwelling older adults.

#### 2.2.1 Participants and Procedure

A total of 263 Croatian‐speaking older adults aged 65 and over were recruited through convenience sampling via Qualtrics survey software between January and May 2025.

Eligibility criteria required participants to be 65 years or older, living independently in Croatia and able to read and understand Croatian sufficiently to complete the questionnaire. Participants were expected to be in generally good physical and cognitive health, with no self‐reported history of dementia, stroke, or other diagnosed neurodegenerative or psychiatric conditions that may impact functional ability. The questionnaire was completed online; therefore, participants also required basic digital literacy and access to the Internet.

Demographic data collected included age, gender, education level, years of schooling, and self‐reported memory problems. Of the total sample, 56 participants completed the questionnaire again after a 2‐week interval for test‐retest reliability assessment. All participants provided informed consent.

#### 2.2.2 Adapted Instrument

The final Croatian version of the Details of Functions of Everyday Life – Revised (DoFEL‐R; Detalji funkcija svakodnevnog života – Revidirano) consisted of 44 items, each rated on a 4‐point scale from 0 (Not applicable) to 3 (Often), with higher scores indicating more significant impairment in daily functioning. Items are grouped across seven domains to capture the frequency of functional difficulties in older adults across varying everyday tasks. The translated and culturally adapted scale is provided in Appendix (A).

The DoFEL is a theory‐driven instrument developed to assess subtle everyday functional changes associated with cognitive decline, particularly in preclinical AD dementia. Unlike traditional IADL scales, which typically provide a global score of task performance [8], the DoFEL‐R deconstructs complex functional tasks into their constituent cognitive components, enabling more precise inference about the memory and cognitive processes underlying functional decline. Additionally, as. [8] noted, a gap in conventional scales is their lack of attention to what specific aspects of a task are impaired. The DoFEL‐R addresses this gap by embedding binding theory, a framework used to understand how information is integrated into memory, into item design.

#### 2.2.3 Data Analysis

Data were analysed using IBM SPSS v25. Four participants with missing data (> 10% of incomplete items) were excluded. Before factor analysis, the assumptions of normality, linearity, and sampling adequacy were assessed to ensure the data were likely to be factorisable.

An exploratory factor analysis (EFA) was conducted using principal axis factoring with oblique rotation. A two‐factor structure corresponding to relational and conjunctive binding was predefined based on the original measure. Given the sample size and preliminary nature of this adaptation study, EFA was selected to explore the underlying structure and assess alignment with the theoretical two‐factor model. Internal consistency was evaluated using Cronbach′s alpha. Additionally, Test‐retest reliability was assessed using intraclass correlation coefficients (ICCs) after a 2‐week interval with 56 participants. All statistical tests were two‐sided with a significance threshold of *p* < 0.05.

## 3 Results

### 3.1 Participant Characteristics

Of the 267 participants who completed the Croatian version of the DoFEL‐R, four were excluded due to missing data, resulting in a final sample of 263 older adults. Participants ranged in age from 65 to 88 years (M = 72.6, SD = 6.1), with approximately 62% identifying as female. Most participants had completed secondary education or higher, with a mean of 13.4 years of formal education (SD = 3.2). Approximately 21% self‐reported experiencing memory difficulties.

### 3.2 Face Validity

Feedback obtained through cognitive interviews (*n* = 4) indicated that participants generally found the items understandable and relevant. Minor wording revisions were made to enhance clarity, particularly in items that reference modern technologies and household roles. No items were reported as confusing or culturally inappropriate, supporting the face validity of the adapted scale.

### 3.3 Exploratory Factor Analysis

The suitability of the data for factor analysis was confirmed. The Kaiser–Meyer–Olkin (KMO) measure of sampling adequacy was 0.81, and Bartlett′s test of sphericity was significant (*χ*²(946) = 2693.24, *p* < 0.001), indicating adequate inter‐item correlations for factor extraction.

Principal axis factoring with oblique rotation revealed a two‐factor solution consistent with the predefined model. Factor 1 (relational binding) accounted for 36.2% of the variance, while Factor 2 (conjunctive binding) explained an additional 21.7%, together accounting for 57.9% of the total variance. Most items loaded clearly onto one of the two factors, with cross‐loadings within acceptable limits (< 0.30). Item loadings ranged from 0.42 to 0.81 across the two factors. Factor 1 (relational binding) included items related to integrating information across people, places, and events, such as “Zaboravlja datum i vrijeme zadataka i dogadaja” (Forgets the date and time of tasks and events). Factor 2 (conjunctive binding) included items that involve combining detailed features or elements within a task, such as “Ima poteškoća s prepoznavanjem lijekova” (Has difficulty recognising medications). These findings align with the theoretical framework underpinning the original DoFEL‐R. A full item‐by‐factor loading table is provided in Appendix B.

### 3.4 Test‐Retest Reliability

Test‐retest reliability was assessed in a subsample of 56 participants who completed the questionnaire twice over a 2‐week interval. Intraclass correlation coefficients (ICCs) indicated excellent stability, with an ICC of 0.91 for the total DoFEL‐R score (95% CI: 0.86–0.95).

### 3.5 Internal Consistency

Cronbach's alpha demonstrated good internal consistency for the scale (*α* = 0.89).

## 4 Discussion

This study presents the translation, cross‐cultural adaptation, and preliminary validation of the Croatian version of the Details of Functions of Everyday Life – Revised (DoFEL‐R), a theory‐driven tool designed to detect subtle functional changes associated with early‐stage Alzheimer's disease (AD). Following the ITC guidelines (2017), a multi‐stage adaptation process ensured linguistic and cultural relevance while preserving the original measure's theoretical foundation.

The adapted scale demonstrated strong face validity, with participants reporting the items as clear, relevant, and appropriate for the Croatian context. EFA supported the predefined two‐factor model, broadly reflecting relational and conjunctive binding, in line with the theoretical basis of the original scale [4]. Internal consistency was high (Cronbach's *α* = 0.89), and test‐retest reliability over a 2‐week interval was excellent (ICC = 0.91). These findings offer initial psychometric support for the Croatian DoFEL‐R as a reliable and conceptually valid measure of early functional decline, with potential clinical utility in identifying individuals at risk of progression from MCI to AD.

While the current findings support the internal structure and reliability of the Croatian DoFEL‐R, future studies are needed to establish criterion validity by correlating scores with established cognitive measures such as the MMSE or RUDAS and by comparing performance across clinically diagnosed groups, including those with MCI or early‐stage AD. Notably, the English version of the DoFEL‐R has demonstrated strong criterion validity in distinguishing individuals with MCI from cognitively healthy older adults [4]. O′Donald et al., 2025), supporting its potential for diagnostic use in other cultural contexts. Additionally, future studies using CFA are needed to formally test the theoretical model and assess measurement invariance across cultural groups.

### 4.1 Limitations

Several limitations should be noted. First, the use of convenience sampling resulted in a sample of relatively well‐educated, digitally literate older adults, which may limit the generalisability of findings to the broader Croatian elderly population, particularly those with lower education levels or limited experience with digital technologies. Second, although participants self‐reported being in good cognitive and physical health, the absence of clinical or cognitive screening raises the possibility that undiagnosed conditions may have influenced responses on the DoFEL‐R. The lack of validation against cognitive screening tools or clinical diagnoses limits our ability to draw conclusions about the diagnostic accuracy of the Croatian DoFEL‐R. However, the English version of the scale has shown promising diagnostic accuracy in differentiating MCI from healthy ageing [4], suggesting a strong foundation for future criterion validation in the Croatian context. Third, the limited sample size restricted the scope of psychometric analyses, including differential item functioning and subgroup comparisons for cross‐cultural validity. Lastly, the study did not incorporate external validity measures, such as neuropsychological assessment or clinical diagnosis, which would be essential for establishing criterion validity.

Further validation in larger and more diverse samples, including those with diverse educational backgrounds, is needed. Future studies should also include objective cognitive measures to evaluate the accuracy of the Croatian version of the DoFEL‐R in detecting cognitive impairment. Longitudinal research examining the predictive validity of the DoFEL‐R to identify individuals at increased risk of cognitive decline is also required before this measure can be reliably used in clinical settings.

## 5 Conclusions

This study provides initial evidence supporting the Croatian DoFEL‐R as a culturally appropriate, theoretically grounded, and psychometrically sound instrument for assessing everyday functional changes in Croatian older adults. By incorporating the principles of memory binding into items, the DoFEL‐R has the potential to capture early functional impairments often missed by traditional tools. Its application may support earlier detection and more timely intervention in AD in Croatian settings and potentially in other similar cultural contexts.

## Author Contributions

**Freddie O'Donald:** conceptualization, methodology, formal analysis, project administration, validation, investigation, writing – original draft, writing – review and editing, visualization. **Mario A Parra:** conceptualization, writing – review and editing, visualization, supervision. **Clara Calia:** visualization, project administration, investigation, validation, writing – review and editing. all authors have read and approved the final version of the manuscript.

## Conflicts of Interest

The authors declare no conflicts of interest.

## Transparency Statement

The lead author (Freddie O'Donald) affirms that this manuscript is an honest, accurate, and transparent account of the study being reported; that no important aspects of the study have been omitted; and that any discrepancies from the study as planned have been explained.

## Data Availability

The data that support the findings of this study are available from the corresponding author upon reasonable request. Freddie O'Donald had full access to all of the data in this study and takes complete responsibility for the integrity of the data and accuracy of the data analysis.

## Appendix 11

Detalji funkcija svakodnevnog života – Revidirano (DoFEL‐R)

Molimo označite odgovarajući broj za svaku stavku prema učestalosti pojavljivanja:

0 = Nije primjenjivo

1 = Nikad

2 = Povremeno

3 = Često

Kupovina i novac

**Table:**

Pitanje	0	1	2	3
Gubi novac ili zaboravlja gdje ga je stavio (R)				
Ima poteškoća s rukovanjem novcem ili korištenjem digitalnih metoda plaćanja pri plaćanju ili primanju ostatka (R)				
Ima poteškoća s prepoznavanjem uobičajenih proizvoda u supermarketima ili trgovinama (C)				
Zaboravlja lokaciju proizvoda kod kuće ili u supermarketima (R)				
Ima poteškoća s pamćenjem potrebnih artikala bez popisa za kupovinu (C)				
Ukupno:				

Predmeti i osobe

**Table:**

Pitanje	0	1	2	3
Ima poteškoća s prepoznavanjem osobnih predmeta (npr. ključevi, odjeća) (C)				
Ima poteškoća s pamćenjem lokacije osobnih predmeta (R)				
Ima poteškoća s prepoznavanjem poznatih predmeta (npr. vrtni alat, knjiga) (C)				
Ima poteškoća s prepoznavanjem lica poznatih osoba (C)				
Ima poteškoća s prisjećanjem imena poznatih osoba (R)				
Ima poteškoća s prisjećanjem imena nedavno upoznatih osoba (R)				
Ima poteškoća s prepoznavanjem kuće novih prijatelja (C)				
Zaboravlja gdje je parkirao automobil (R)				
Ima poteškoća s prepoznavanjem vlastitog automobila (npr. miješa ga sa sličnim automobilom) (C)				
Ukupno:				

Tehnologija i komunikacija

**Table:**

Pitanje		0	1	2	3
Zaboravlja nedavno naučene telefonske brojeve (C)					
Ima poteškoća s korištenjem:	Mobitela (C)				
	Aplikacija za komunikaciju (npr. Viber, WhatsApp, Messenger) (C)				
Ima poteškoća s prepoznavanjem vlastitog mobitela (C)					
Ima poteškoća s korištenjem bankomata (R)					
Ima poteškoća s pamćenjem PIN koda različitih bankovnih kartica (R)					
Ima poteškoća s praćenjem radnje u TV programima (R)					
Ima poteškoća s prepoznavanjem i/ili korištenjem digitalne komunikacije (e‐mail, aplikacije za dopisivanje) (R)					
Ukupno:					

Prijevoz i mobilnost

**Table:**

Pitanje	0	1	2	3
Zaboravlja osnovne radnje pri vožnji (npr. kako uključiti svjetla na automobilu) ili ima poteškoća s korištenjem javnog prijevoza (R)				
Ima poteškoća s prepoznavanjem prometnih znakova ili snalaženjem u prometu (npr. GPS, ulične oznake) (C)				
Ima poteškoća s vožnjom na poznatim lokacijama (R)				
Ima poteškoća s vožnjom na nepoznatim lokacijama (R)				
Ima poteškoća s korištenjem orijentira pri hodanju na poznata mjesta (R)				
Ima poteškoća s korištenjem orijentira pri hodanju na nepoznata mjesta (R)				
Ukupno:				

Kućanski poslovi

**Table:**

Pitanje	0	1	2	3
Ima poteškoća s korištenjem dobro poznatih ili prethodno korištenih električnih uređaja (C)				
Zaboravlja lokaciju električnih uređaja kod kuće (R)				
Koristi pogrešne kuhinjske alate ili ima poteškoća s tradicionalnim načinima kuhanja (R)				
Zaboravlja imena sastojaka tijekom kuhanja (R)				
Ukupno:				

Posao i društveni život

**Table:**

Pitanje	0	1	2	3
Zaboravlja imena osoba u poslovnim ili društvenim situacijama (R)				
Zaboravlja datum i vrijeme zadataka i događaja (R)				
Ima poteškoća s određivanjem prioriteta i pamćenjem važnih zadataka (R)				
Ima poteškoća s pravilnim oblačenjem i organiziranjem za sastanke (R)				
Zaboravlja aktivnosti u svojoj uobičajenoj dnevnoj rutini (C)				
Ima poteškoća s prepoznavanjem bliskih članova obitelji ili osoba s kojima redovito komunicira (C)				
Ukupno:				

Zdravlje i stil života

**Table:**

Pitanje	0	1	2	3
Ima poteškoća s prepoznavanjem lijekova (npr. za što određena tableta služi) ili tradicionalnih lijekova (C)				
Zaboravlja gdje su lijekovi spremljeni (R)				
Ima poteškoća s prepoznavanjem promjena u vlastitom fizičkom stanju (težina, energija) (C)				
Zaboravlja redovne slobodne aktivnosti (šetnje, čitanje) (C)				
Povlači se iz uobičajenih slobodnih aktivnosti (C)				
Ukupno:				

Ukupni rezultat

Ukupno Relacijske funkcije (R) (Ukupno 24) ____ od _____

Ukupno Konjunktivne funkcije (C) (Ukupno 20) ____ od _____

Izračun standardiziranih rezultata:

1. Najprije isključite stavke označene kao “nije primjenjivo”.
2. Zbrojite odgovore za preostale stavke kako biste dobili ukupni rezultat.
3. Izbrojite uključene stavke i pomnožite ih s 3 kako biste dobili maksimalni rezultat.
4. Podijelite ukupni rezultat s maksimalnim rezultatom kako biste dobili standardizirani rezultat između 0 i 1.

*Veći rezultati ukazuju na veći stupanj oštećenja u svakodnevnom funkcioniranju. Trenutno radimo na razvijanju graničnih vrijednosti za određivanje različitih razina oštećenja*.

## Appendix 12

**Table:**

DoFEL‐R Items (Croatian)	Theoretical factor	Factor 1	Factor 2	Communalities
*Gubi novac ili zaboravlja gdje ga je stavio*	Relational	0.74		0.66
*Ima poteškoća s rukovanjem novcem ili korištenjem digitalnih metoda plaćanja pri plaćanju ili primanju ostatka*	Relational	0.81		0.69
*Ima poteškoća s prepoznavanjem uobičajenih proizvoda u supermarketima ili trgovinama*	Conjunctive		0.76	0.72
*Zaboravlja lokaciju proizvoda kod kuće ili u supermarketima*	Relational	0.68		0.58
*Ima poteškoća s pamćenjem potrebnih artikala bez popisa za kupovinu*	Conjunctive		0.79	0.61
*Ima poteškoća s prepoznavanjem osobnih predmeta (npr. ključevi, odjeća)*	Conjunctive		0.73	0.66
*Ima poteškoća s pamćenjem lokacije osobnih predmeta*	Relational	0.69		0.59
*Ima poteškoća s prepoznavanjem poznatih predmeta (npr. vrtni alat, knjiga)*	Conjunctive		0.77	0.7
*Ima poteškoća s prepoznavanjem lica poznatih osoba*	Conjunctive		0.68	0.55
*Ima poteškoća s prisjećanjem imena poznatih osoba*	Relational	0.66		0.53
*Ima poteškoća s prisjećanjem imena nedavno upoznatih osoba*	Relational	0.64		0.52
*Ima poteškoća s prepoznavanjem kuće novih prijatelja*	Conjunctive		0.65	0.58
*Zaboravlja gdje je parkirao automobil*	Relational	0.61		0.5
*Ima poteškoća s prepoznavanjem vlastitog automobila (npr. miješa ga sa sličnim automobilom)*	Conjunctive		0.75	0.69
*Zaboravlja nedavno naučene telefonske brojeve*	Conjunctive		0.72	0.6
*Ima poteškoća s korištenjem: Mobitela*	Conjunctive		0.8	0.67
*Ima poteškoća s korištenjem aplikacija za komunikaciju (npr. Viber, WhatsApp, Messenger)*	Conjunctive		0.81	0.68
*Ima poteškoća s prepoznavanjem vlastitog mobitela*	Conjunctive		0.78	0.66
*Ima poteškoća s korištenjem bankomata*	Relational	−0.45		0.51
*Ima poteškoća s pamćenjem PIN koda različitih bankovnih kartica*	Relational	0.61		0.48
*Ima poteškoća s praćenjem radnje u TV programima*	Relational	0.6	0.32	0.49
*Ima poteškoća s prepoznavanjem i/ili korištenjem digitalne komunikacije (e‐mail, aplikacije za dopisivanje)*	Relational	0.73		0.63
*Zaboravlja osnovne radnje pri vožnji (npr. kako uključiti svjetla na automobilu) ili ima poteškoća s korištenjem javnog prijevoza*	Relational	0.79		0.68
*Ima poteškoća s prepoznavanjem prometnih znakova ili snalaženjem u prometu (npr. GPS, ulične oznake)*	Conjunctive		0.66	0.57
*Ima poteškoća s vožnjom na poznatim lokacijama*	Relational	0.77		0.65
*Ima poteškoća s vožnjom na nepoznatim lokacijama*	Relational	0.78		0.66
*Ima poteškoća s korištenjem orijentira pri hodanju na poznata mjesta*	Relational	0.75		0.61
*Ima poteškoća s korištenjem orijentira pri hodanju na nepoznata mjesta*	Relational	0.76		0.63
*Ima poteškoća s korištenjem dobro poznatih ili prethodno korištenih električnih uređaja*	Conjunctive		0.71	0.59
*Zaboravlja lokaciju električnih uređaja kod kuće*	Relational	0.62		0.5
*Koristi pogrešne kuhinjske alate ili ima poteškoća s tradicionalnim načinima kuhanja*	Relational	0.64		0.51
*Zaboravlja imena sastojaka tijekom kuhanja*	Relational	−0.49	0.35	0.45
*Zaboravlja imena osoba u poslovnim ili društvenim situacijama*	Relational	0.73		0.6
*Zaboravlja datum i vrijeme zadataka i događaja*	Relational	0.81		0.67
*Ima poteškoća s određivanjem prioriteta i pamćenjem važnih zadataka*	Relational	0.77		0.65
*Ima poteškoća s pravilnim oblačenjem i organiziranjem za sastanke*	Relational	0.69		0.58
*Zaboravlja aktivnosti u svojoj uobičajenoj dnevnoj rutini*	Conjunctive		0.69	0.55
*Ima poteškoća s prepoznavanjem bliskih članova obitelji ili osoba s kojima redovito komunicira*	Conjunctive		0.74	0.6
*Ima poteškoća s prepoznavanjem lijekova (npr. za što određena tableta služi) ili tradicionalnih lijekova*	Conjunctive		0.78	0.64
*Zaboravlja gdje su lijekovi spremljeni*	Relational	0.67		0.53
*Ima poteškoća s prepoznavanjem promjena u vlastitom fizičkom stanju (težina, energija)*	Conjunctive		0.81	0.66
*Zaboravlja redovne slobodne aktivnosti (šetnje, čitanje)*	Conjunctive		0.8	0.64
*Povlači se iz uobičajenih slobodnih aktivnosti*	Conjunctive		0.79	0.63
